# Decoding high Gonadotropin-releasing hormone pulsatility: a role for GnRH receptor coupling to the cAMP pathway?

**DOI:** 10.3389/fendo.2012.00107

**Published:** 2012-08-31

**Authors:** Joëlle Cohen-Tannoudji, Charlotte Avet, Ghislaine Garrel, Raymond Counis, Violaine Simon

**Affiliations:** Equipe Physiologie de l’Axe Gonadotrope, Unité de Biologie Fonctionnelle et Adaptative, CNRS-EAC 4413, Sorbonne Paris Cité, Université Paris Diderot-Paris 7Paris, France

**Keywords:** pituitary, GnRH pulsatile pattern, GnRH receptor, gonadotrope cell signaling, cAMP pathway

## Abstract

The gonadotropin-releasing hormone (GnRH) pulsatile pattern is critical for appropriate regulation of gonadotrope activity but only little is known about the signaling mechanisms by which gonadotrope cells decode such pulsatile pattern. Here, we review recent lines of evidence showing that the GnRH receptor (GnRH-R) activates the cyclic AMP (cAMP) pathway in gonadotrope cells, thus ending a long-lasting controversy. Interestingly, coupling of GnRH-R to the cAMP pathway as well as induction of nitric oxide synthase 1 (NOS1) or follistatin through this signaling pathway take place preferentially under high GnRH pulsatility. The preovulatory surge of GnRH *in vivo* is indeed associated with an important increase of pituitary cAMP and NOS1 expression levels, both being markedly inhibited by treatment with a GnRH antagonist. Altogether, this suggests that due to its atypical structure and desensitization properties, the GnRH-R may continue to signal through the cAMP pathway under conditions inducing desensitization for most other receptors. Such a mechanism may contribute to decode high GnRH pulsatile pattern and enable gonadotrope cell plasticity during the estrus cycle.

## INTRODUCTION

Reproduction success depends on the coordinated synthesis and release of several hormones from the hypothalamus–pituitary–gonadal axis. Mammalian gonadotropin-releasing hormone (known as GnRH 1) mediates brain control of reproductive activity and, as a major actor of gonadotrope axis, has received considerable attention. The neurohormone GnRH is a decapeptide produced by a small number of scattered hypothalamic neurons whose cell bodies are primarily located in the preoptic area and basal hypothalamus. GnRH neurons project to the median eminence and secrete GnRH in a pulsatile fashion into the hypophysial portal vessels through which GnRH is transported to the anterior pituitary gland. The release of GnRH by hypothalamic neurons is influenced by numerous external and internal factors acting through central nervous pathways. The pattern of pulsatile GnRH secretion thus varies widely in both males and females depending on the reproductive status. GnRH acts *via* its receptor specifically expressed in gonadotrope cells to stimulate both synthesis and exocytosis of the two gonadotropins, luteinizing hormone (LH) and follicle-stimulating hormone (FSH). LH and FSH will in turn act on the gonads in a coordinated manner to initiate sexual maturation and regulate gonadal steroidogenesis and gametogenesis in both sexes. Gonadotrope hormones are complex endocrine signals constituted of non-covalently associated glycoprotein dimers. Each gonadotropin is composed of an alpha glycoprotein subunit common to LH, FSH, thyrotropin (TSH) and placental choriogonadotropin (for a few species) and a unique beta subunit.

The GnRH pulsatile pattern is critical for appropriate regulation of LH and FSH synthesis and secretion. Indeed, intermittent stimulation *in vivo* or *in vitro* that mimics the physiological pulsatile release of GnRH efficiently stimulates the secretion of gonadotropins. In contrast, a continuous pattern leads to desensitization of gonadotrope cells and this has been exploited by clinicians to suppress gonadotropin secretion (Lahlou et al., 1987). Furthermore, pulsatility of GnRH varies throughout the ovarian cycle and accounts for the differential secretion of LH and FSH. At mid-cycle, during proestrus, an abrupt and massive increase in GnRH pulsatility is responsible for gonadotropin surge and ovulation. Only little is known about the signaling mechanisms by which the pituitary gonadotrope cells decode GnRH pulse pattern. The aim of this article is to review the current knowledge on GnRH receptor (GnRH-R) coupling to the cyclic AMP (cAMP) signaling pathway in order to highlight its potential role in decoding high GnRH pulsatility.

## COUPLING OF THE GnRH RECEPTOR TO THE cAMP SIGNALING PATHWAY

GnRH binds to a receptor belonging to the G protein-coupled receptor (GPCR) family with seven transmembrane domains connected by extracellular and intracellular loops. Agonist binding is mainly associated with a rapid Gq/11-mediated increase in phospholipase Cβ (PLCβ) activity, which will in turn initiate a wide array of signaling events. Hydrolysis of phosphatidylinositol 4,5-bisphosphate (PIP2) results in the formation of diacylglycerol (DAG) and inositol trisphosphate (IP3). Rapid formation of IP3 induces calcium release from intracellular stores and, together with GnRH-stimulated calcium influx, accounts for calcium oscillations that trigger gonadotropin exocytosis. Elevation of calcium also activates the nitric oxide synthase (NOS) cascade (NOS1/NO/soluble guanylate cyclase), resulting in a rapid increase of cyclic GMP (cGMP) levels (Naor et al., 1980; Lozach et al., 1998). GnRH-induced DAG formation activates protein kinase C (PKC) isoforms belonging to the three known families of PKC (conventional, novel, and atypical), which mediate notably activation of mitogen-activated protein kinases (MAPK) cascades. PKC and MAPK signaling are crucial for the regulation of gonadotropin subunit gene expression (Thackray et al., 2009). Following a short time lag, GnRH also activates phospholipase D (PLD) and phospholipase A2 (PLA2). PKC favors the coupling of GnRH-R to PLD leading to a sustained second wave of DAG that may contribute to maintain PKC activation during prolonged stimulation by GnRH (Zheng et al., 1994). GnRH-mediated PLA2 activation generates arachidonic acid and its lipoxygenase products that have been implicated in GnRH-induced gonadotropin synthesis and release (Naor, 2009). The GnRH-R thus activates a wide array of signaling entities to regulate gonadotropin synthesis and release.

**FIGURE 1:**
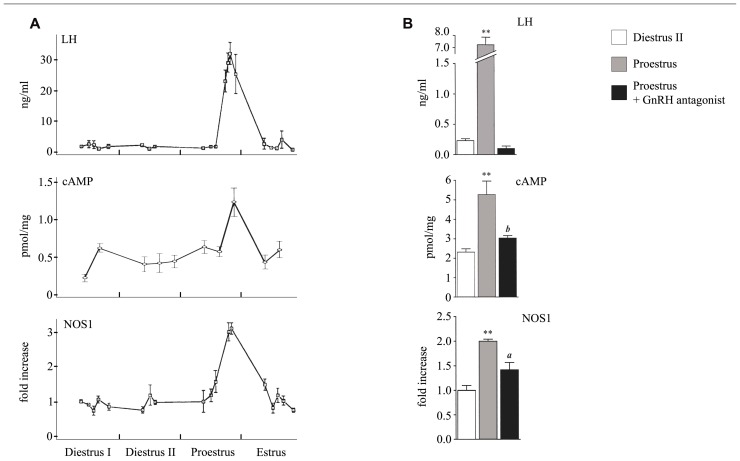
** Profiles of serum LH and pituitary cAMP and NOS1 levels during the proestrus and following administration of a GnRH antagonist.** **(A)** Serum LH, pituitary cAMP, and pituitary NOS1 levels were determined by radioimmunoassay, ELISA, and western blot analysis, respectively. **(B)** Effect of an *in vivo* treatment with the GnRH antagonist, antarelix.^ a^*P* <0.05 and ^ b^*P* <0.01 compared with proestrus, **P <0.01 compared with diestrus II. Modified from Kimura et al. (1980), Lozach et al. (1998), and Garrel et al. (2010).

It has been clearly established that the cAMP/protein kinase A (PKA) pathway is essential for gonadotrope function. Indeed, cAMP analogs mimic most of the effects of GnRH as they enhance the release of newly synthesized LH and the expression of several key genes including those encoding LHβ and α subunits as well as GnRH-R and NOS1 (Starzec et al., 1989; Garrel et al., 2002; Horton and Halvorson, 2004). However, the ability of GnRH to induce cAMP production in gonadotrope cells as well as the involvement of cAMP in GnRH action has long remained debated. Early observation of Borgeat et al. (1975) showed that a prolonged exposure (3 h) of rat hemipituitaries to GnRH stimulates cAMP accumulation and this observation was confirmed soon after by others (Naor et al., 1975). Since then, studies performed on dispersed rat pituitary cell cultures did not evidence any cAMP production or stimulation of adenylyl-cyclase (AC) activity in response to GnRH (Theoleyre et al., 1976; Conn et al., 1979). In 1989, the pituitary AC-activating polypeptide (PACAP) was discovered based on its ability to strongly activate AC in rat pituitary cells (Miyata et al., 1989). This probably contributed to lower the interest paid to GnRH-R signaling through the cAMP pathway. Furthermore, no GnRH-induced cAMP production could be substantiated on the first gonadotrope cell line that was established in the same period of time, the αT3-1 cell line (Horn et al., 1991). Accordingly, using photolabeling experiments, Grosse et al. (2000) argued for the exclusive coupling of GnRH-R to Gαq/11 in αT3-1 cells. Up to the early 2000s, the coupling of GnRH-R to the cAMP pathway in gonadotrope cells thus remained elusive. The ability of the mammalian GnRH-R to stimulate the cAMP pathway was however reported in several heterologous systems such as GH_3_, COS-7, or HeLa cells (Arora et al., 1998; Lin et al., 1998; Oh et al., 2005), thus reinforcing the interest for such coupling. The key to moving forward was the development of novel models and technologies. The ability of GnRH to induce cAMP accumulation in gonadotrope cells was demonstrated by two different groups including ours (Liu et al., 2002; Lariviere et al., 2007) using the gonadotrope cell line LβT2, which is more fully differentiated than the αT3-1 cell line. cAMP accumulation was evidenced by biochemical and enzyme-linked immunosorbent assays (ELISAs) and was specifically induced by GnRH-R activation as shown by competition with a GnRH antagonist. Further analysis revealed that different mechanisms contribute to GnRH-R-induced cAMP accumulation. Indeed, based on photolabeling experiments and on cell-permeable peptides that uncouple the receptor from Gαs, it was shown that GnRH-R activates cAMP production *via* Gs recruitment (Liu et al., 2002). Additionally, we demonstrated, using selective pharmacological inhibitors as well as dominant negative mutants of PKC isoforms, that novel PKCδ and ε mediate GnRH-R activation of the cAMP pathway (Lariviere et al., 2007). In both studies, GnRH-induced increase in cAMP levels exhibited atypical features. Indeed, GnRH-induced maximal accumulation of cAMP levels was observed only after a lag of time of approximately 30 min and was delayed as compared to response to PACAP (Lariviere et al., 2007) or to the beta-adrenergic receptor agonist, isoproterenol (J. Cohen-Tannoudji, personal communication). Furthermore, maximal cAMP accumulation was weaker and this may explain why GnRH-R coupling to this signaling pathway has long remained controversial. This prompted us to reevaluate the coupling of GnRH-R to the cAMP signaling pathway in αT3-1 cells using a recently developed technology. As reported previously by Horn et al. (1991), we did not detect any significant increase in cAMP levels by ELISAs when cells were challenged with a GnRH agonist in contrast to response to PACAP or forskolin stimulation. We then took advantage of a sensitive technology based on the use of a cAMP biosensor (pGloSensor^TM^-22F cAMP Plasmid). αT3-1 cells were transfected with a plasmid encoding an engineered cAMP sensitive luciferase, which becomes active upon cAMP binding. Transfected cells were challenged with a GnRH agonist and the luminescence intensity was measured in real time in living cells. This strategy allowed us to demonstrate for the first time that GnRH-R couples to the cAMP pathway in αT3-1 cells (Avet et al., 2012). Indeed, GnRH dose-dependently increased luciferase activity reflecting cAMP production with an EC_50_ in the range of 1 nM and the increase was significantly inhibited by co-incubation with the GnRH antagonist, antide. Once the ability of GnRH to stimulate cAMP production was established in both gonadotrope cell lines, our efforts turned toward trying to understand whether this could also occurred in native gonadotrope cells. A massive pituitary cAMP level increase has been reported long ago to occur during the proestrus afternoon of the rat estrus cycle, coincidently with the GnRH preovulatory surge (Kimura et al., 1980). However, despite such temporal coincidence, the precise contribution of GnRH has never been elucidated. We reevaluated this contribution *in vivo* by administrating a potent GnRH antagonist, the ganirelix, to females at the evening of the diestrus II (Garrel et al., 2010). The marked decrease in pituitary cAMP levels provided evidence for a predominant role of GnRH in mediating the proestrus-specific rise of cAMP (**Figure 1**).

## THE ATYPICAL STRUCTURE OF GnRH RECEPTOR IS ASSOCIATED WITH RESISTANCE TO RAPID DESENSITIZATION

All mammalian pituitary GnRH-R (named type I GnRH-R) exhibit a striking structural feature, which is the lack of a cytoplasmic carboxyl-terminal tail. This carboxyl-terminal tail is present in all other GPCR including GnRH-R from non-mammalian vertebrates and is a target for kinases such as GRK (GPCR kinase). GRK-mediated phosphorylation promotes the binding of β-arrestins, which not only uncouple receptors from G proteins resulting in desensitization but also target many GPCR for internalization in clathrin-coated vesicles (Ferguson, 2001). Given the unique absence of cytoplasmic carboxyl-terminal tail, the initial rate of inositol phosphate accumulation is linear upon 10 min of GnRH stimulation showing that GnRH-R is resistance to rapid desensitization (Davidson et al., 1994). Accordingly, mammalian GnRH-R is not phosphorylated upon agonist stimulation and does not recruit β-arrestins, in contrast to non-mammalian GnRH-R (Willars et al., 1999; McArdle et al., 2002). Extensive analysis of GnRH-R internalization has been performed using biochemical approaches and fluorescent or radiolabeled GnRH in gonadotrope and heterologous cells. These studies indicated that mammalian GnRH-R is poorly internalized as compared to non-mammalian GnRH-R and that addition of a functional intracellular carboxyl-terminal tail to the receptor significantly increased internalization rates and rapid desensitization. Whereas these studies were rather measuring ligand internalization, investigations conducted by Millar’s group, using an epitope-tagged receptor allowed to trace the receptor itself. These studies established that GnRH-R exhibits a low level of constitutive internalization and does not undergo rapid agonist-dependent internalization as compared to non-mammalian GnRH or the TSH-releasing hormone receptors (Pawson et al., 2008). The refractory state of gonadotrope cells under a sustained GnRH challenge is thus believed to occur through desensitization mechanism affecting downstream signaling entities such as Gαq/11, PKC isoforms, or IP3 receptors (Willars et al., 2001; Liu et al., 2003) rather than the receptor itself. Consequently, mammalian GnRH-R has the unique property amongst GPCR to face prolonged activation by its ligand.

## MECHANISMS CONTRIBUTING TO GnRH-R COUPLING TO THE cAMP PATHWAY

One intriguing property of GnRH-R coupling to the cAMP pathway in LβT2 cells is that it appears to be dependent on GnRH stimulation pattern. We, indeed, observed that an intermittent stimulation of cells with GnRH, known to stimulate calcium release or MAPK activation, was ineffective in inducing cAMP accumulation (Lariviere et al., 2007). Levels of cAMP were significantly increased only under sustained stimulation of GnRH-R suggesting that this pathway may be preferentially recruited under high frequency of GnRH pulses, in agreement with the proestrus-specific elevation of pituitary cAMP levels during rat ovarian cycle (Kimura et al., 1980; Garrel et al., 2010). This shows that GnRH-R maintains cAMP pathway activation under stimulations inducing desensitization of most GPCR. This atypical feature was recently confirmed using fluorescence resonance energy transfer reporters in LβT2 cells in which high pulse frequency of GnRH desensitized DAG and calcium but not cAMP activation (Tsutsumi et al., 2010).

Only few genes regulated by GnRH through the cAMP/PKA signaling pathway have been identified so far. Among them are those encoding follistatin, Nur77, PACAP, and NOS1 (Winters et al., 2007; Hamid et al., 2008; Grafer et al., 2009; Mutiara et al., 2009; Garrel et al., 2010). Interestingly, analysis of how GnRH regulates two of these genes has revealed that they are preferentially induced under high GnRH pulse frequency. Follistatin transcript and protein are increased by GnRH administered continuously or at fast frequency pulses either *in vivo* in rat or in a perifused pituitary cells (Besecke et al., 1996) and not at slow GnRH pulse frequencies. Because follistatin binds to and neutralizes activin, stimulation of FSHβ by GnRH, which requires activin is blocked. This is believed to explain why expression of FSHβ is preferentially stimulated under low frequency of GnRH pulses. In rat anterior pituitary gland, NOS1 is expressed specifically in gonadotrope and folliculostellate cells. Our group has demonstrated from experiments performed *in vivo* in rats and *in vitro* that NOS1 expression is regulated by GnRH through the cAMP pathway (Garrel et al., 1998; Lozach et al., 1998). This was shown in particular by taking advantage of a cell-permeant PKA inhibitor (PKI) peptide delivery in primary cultures of rat pituitary cells (Garrel et al., 2010). Interestingly, we observed that NOS1 is preferentially induced by prolonged GnRH treatment. Indeed, NOS1 expression remained at high levels for at least 48 h after treatment of rats with a long-lasting GnRH agonist, whereas, at the same time, LH and FSH secretion was desensitized. Accordingly, we demonstrated in perifused rat pituitary cells that NOS1 is not induced by GnRH delivered at a frequency of one pulse per hour, although this frequency is able to trigger a massive LH release. In contrast, NOS1 was maximally induced by a continuous GnRH stimulation, which suppressed gonadotropin release. Such a result is in agreement with *in vivo* data showing a marked increase of NOS1 expression at proestrus during the preovulatory GnRH surge, which is characterized by a very high GnRH pulse frequency (Lozach et al., 1998; **Figure 1**). Interestingly, treatment with the GnRH antagonist ganirelix not only blocked the proestrus-specific cAMP increase (see above) but also significantly reduced the rise of NOS1 expression levels (Garrel et al., 2010; **Figure 1**) further supporting the physiological relevance of GnRH-R coupling to the cAMP pathway. Altogether, experiments reported here, suggest that under conditions of high GnRH pulsatility, GnRH-R, which is atypically maintained at the cell surface, still interacts with its intracellular machinery and activates the cAMP/PKA pathway. This initiates the expression of a new set of target proteins among which are NOS1 and follistatin. The role of GnRH-dependent NOS1 induction at proestrus remains to be determined. The specific increase of NOS1 expression leads to a concomitant huge increase of pituitary cGMP levels at proestrus (Lozach et al., 1998). cGMP may regulate some cyclic nucleotide-gated channels that we have characterized in pituitary gonadotrope cells (J. Cohen-Tannoudji, personal communication) and this is under investigation.

How is the preferential coupling of GnRH-R to the cAMP pathway selected? This question is still unresolved. GnRH-R is constitutively localized in low-density membrane microdomains such as rafts (Navratil et al., 2003). Intense stimulation by GnRH may alter the signaling platform associated with GnRH-R in rafts and hence favor receptor coupling to the cAMP pathway. Another potential mechanism may be related to GPCR-interacting proteins (GIP) that interact through receptor intracellular domains to regulate signaling efficacy and specificity (Bockaert et al., 2004). The idea that GIP regulate GnRH-R signaling is suggested by the fact that introduction in αT3-1 cells of synthetic peptides corresponding to intracellular domains of mammalian GnRH-R increases GnRH-R coupling to the inositol phosphate pathway (Shacham et al., 2005). We very recently identified a protein, the protein SET, as the first direct interacting partner of GnRH-R. Using cAMP and calcium biosensors in αT3-1 living cells combined with small interfering RNA directed against SET, we showed that SET significantly enhances GnRH-R coupling to the cAMP pathway. The mechanisms contributing to SET recruitment and the potential regulatory roles of SET on gonadotrope function are under current investigation.

## CONCLUSION

An important question in the field of reproductive endocrinology is to understand how the GnRH pulsatile pattern is decoded by pituitary gonadotrope cells. Due to its atypical structure and desensitization properties, GnRH-R and some of its signaling mechanisms are continuing to respond under massive GnRH stimulations. Such stimulations of gonadotrope cells by GnRH thus do not lead to general cell desensitization since some genes are induced in response to GnRH at high pulse frequencies. Variation in GnRH pulse profiles may lead to preferential activation of different signaling networks and there is now evidence that the cAMP/PKA pathway contributes to the decoding of high GnRH pulsatility. This emphasizes the unique feature of gonadotrope cells, which maintain part of their functional response under conditions of stimulation inducing desensitization of most GPCR. During the ovarian cycle, the GnRH preovulatory surge lasts much longer than the LH surge (Caraty et al., 2002). Maintenance of some gonadotrope responsiveness while gonadotropins are no longer secreted may contribute to gonadotrope cell plasticity during the estrus cycle. Identifying the mechanisms directing the preferential coupling of GnRH-R to the cAMP pathway is an exciting challenge. Insight into GnRH-R interacting partners and into the dynamics of their interactions with the receptor will undoubtedly help to better understand the plasticity of gonadotrope cell signaling.

## Conflict of Interest Statement

The authors declare that the research was conducted in the absence of any commercial or financial relationships that could be construed as a potential conflict of interest.

## References

[B1] Arora K. K. Krsmanovic L. Z. Mores N. O’Farrell H. Catt K. J. (1998). Mediation of cyclic AMP signaling by the first intracellular loop of the gonadotropin-releasing hormone receptor. *J. Biol. Chem.* 273 25581–25586 9748222 10.1074/jbc.273.40.25581

[B2] Avet C. Garrel G. Denoyelle C. Laverrière J. N. Counis R. Cohen-Tannoudji J. Simon V. (2012). “First identification of a direct interacting partner of the GnRH receptor, the protein SET. Differential impact on coupling to cAMP and calcium pathways,” in *Keystone Symposia’s Meeting* Banff, Canada, February 17–22 (Abstract 105)

[B3] Besecke L. M. Guendner M. J. Schneyer A. L. Bauer-Dantoin A. C. Jameson J. L. Weiss J. (1996). Gonadotropin-releasing hormone regulates follicle-stimulating hormone-beta gene expression through an activin/follistatin auto-crine or paracrine loop. *Endo-crinology* 137 3667–3673 10.1210/endo.137.9.8756531 8756531

[B4] Bockaert J. Fagni L. Dumuis A. Marin P. (2004). GPCR interacting proteins (GIP). *Pharmacol. Ther.* 103 203–221 15464590 10.1016/j.pharmthera.2004.06.004

[B5] Borgeat P. Garneau P. Labrie F. (1975). Calcium requirement for stimulation of cyclic AMP accumulation in anterior pituitary gland by LH-RH. *Mol. Cell. Endocrinol.* 2 117–124 168101 10.1016/0303-7207(75)90053-2

[B6] Caraty A. Delaleu B. Chesneau D. Fabre-Nys C. (2002). Sequential role of e2 and GnRH for the expression of estrous behavior in ewes. *Endocrinology* 143 139–145 11751602 10.1210/endo.143.1.8605

[B7] Conn P. M. Morrell D. V. Dufau M. L. Catt K. J. (1979). Gonadotropin-releasing hormone action in cultured pituicytes: independence of luteinizing hormone release and adenosine 3′,5′- monophosphate production. *Endocrinology* 104 448–453 221178 10.1210/endo-104-2-448

[B8] Davidson J. S. Wakefield I. K. Millar R. P. (1994). Absence of rapid desensitization of the mouse gonadotropin-releasing hormone receptor. *Biochem. J*. 300(Pt 2) 299–302 8002931 10.1042/bj3000299 PMC1138161

[B9] Ferguson S. S. (2001). Evolving concepts in G protein-coupled receptor endocytosis: the role in receptor desensitization and signaling. *Pharmacol. Rev*. 53 1–24 11171937

[B10] Garrel G. Lerrant Y. Siriostis C. Berault A. Magre S. Bouchaud C. Counis R. (1998). Evidence that gonadotropin-releasing hormone stimulates gene expression and levels of active nitric oxide synthase type I in pituitary gonadotrophs, a process altered by desensitization and, indirectly, by gonadal steroids. *Endocrinology* 139 2163–2170 9529006 10.1210/endo.139.4.5890

[B11] Garrel G. Lozach A. Bachir L. K. Laverriere J. N. Counis R. (2002). Pituitary adenylate cyclase-activating polypeptide stimulates nitric-oxide synthase type I expression and potentiates the cGMP response to gonadotropin-releasing hormone of rat pituitary gonadotrophs. *J. Biol. Chem.* 277 46391–46401 12244042 10.1074/jbc.M203763200

[B12] Garrel G. Simon V. Thieulant M. L. Cayla X. Garcia A. Counis R. Cohen-Tannoudji J. (2010). Sustained gonadotropin-releasing hormone stimulation mobilizes the cAMP/PKA pathway to induce nitric oxide synthase type 1 expression in rat pituitary cells in vitro and in vivo at proestrus. *Biol. Reprod*. 82 1170–1179 20181617 10.1095/biolreprod.109.082925

[B13] Grafer C. M. Thomas R. Lambrakos L. Montoya I. White S. Halvorson L. M. (2009). GnRH stimulates expression of PACAP in the pituitary gonadotropes via both the PKA and PKC signaling systems. *Mol. Endocrinol.* 23 1022–1032 19342443 10.1210/me.2008-0477 PMC2703603

[B14] Grosse R. Schmid A. Schoneberg T. Herrlich A. Muhn P. Schultz G. Gudermann T. (2000). Gonadotropin-releasing hormone receptor initiates multiple signaling pathways by exclusively coupling to G(q/11) proteins. *J. Biol. Chem.* 275 9193–9200 10734055 10.1074/jbc.275.13.9193

[B15] Hamid T. Malik M. T. Millar R. P. Kakar S. S. (2008). Protein kinase A serves as a primary pathway in activation of Nur77 expression by gonadotropin-releasing hormone in the LbetaT2 mouse pituitary gonadotroph tumor cell line. *Int. J. Oncol*. 33 1055–1064 18949369

[B16] Horn F. Bilezikjian L. M. Perrin M. H. Bosma M. M. Windle J. J. Huber K. S. Blount A. L. Hille B. Vale W. Mellon P. L. (1991). Intracellular responses to gonadotropin-releasing hormone in a clonal cell line of the gonadotrope lineage. *Mol. Endocrinol.* 5 347–355 1653891 10.1210/mend-5-3-347

[B17] Horton C. D. Halvorson L. M. (2004). The cAMP signaling system regulates LHbeta gene expression: roles of early growth response protein-1, SP1 and steroidogenic factor-1. *J. Mol. Endocrinol*. 32 291–306 14766009 10.1677/jme.0.0320291

[B18] Kimura F. Kawakami M. Nakano H. McCann S. M. (1980). Changes in adenosine 3′,5′-monophosphate and guanosine 3′,5′-monophosphate concentrations in the anterior pituitary and hypothalamus during the rat estrous cycle and effects of administration of sodium pentobarbital in proestrus. *Endocrinology* 106 631–635 6243544 10.1210/endo-106-2-631

[B19] Lahlou N. Roger M. Chaussain J. L. Feinstein M. C. Sultan C. Toublanc J. E. Schally A. V. Scholler R. (1987). Gonadotropin and alpha-subunit secretion during long term pituitary suppression by D-Trp6-luteinizing hormone-releasing hormone microcapsules as treatment of precocious puberty. *J. Clin. Endocrinol. Metab.* 65 946–953 2959680 10.1210/jcem-65-5-946

[B20] Lariviere S. Garrel G. Simon V. Soh J. W. Laverriere J. N. Counis R. Cohen-Tannoudji J. (2007). Gonadotropin-releasing hormone couples to 3′,5′-cyclic adenosine-5′-monophosphate pathway through novel protein kinase Cdelta and -epsilon in LbetaT2 gonadotrope cells. *Endocrinology* 148 1099–1107 17185372 10.1210/en.2006-1473

[B21] Lin X. Janovick J. A. Conn P. M. (1998). Mutations at the consensus phosphorylation sites in the third intracellular loop of the rat gonadotropin-releasing hormone receptor: effects on receptor ligand binding and signal transduction. *Biol. Reprod*. 59 1470–1476 9828194 10.1095/biolreprod59.6.1470

[B22] Liu F. Austin D. A. Webster N. J. (2003). Gonadotropin-releasing hormone-desensitized LbetaT2 go-nadotrope cells are refractory to acute protein kinase C, cyclic AMP, and calcium-dependent signaling. *Endocrinology* 144 4354–4365 12960037 10.1210/en.2003-0204

[B23] Liu F. Usui I. Evans L. G. Austin D. A. Mellon P. L. Olefsky J. M. Webster N. J. (2002). Involvement of both G(q/11) and G(s) proteins in gonadotropin-releasing hormone receptor-mediated signaling in L beta T2 cells. *J. Biol. Chem.* 277 32099–32108 12050161 10.1074/jbc.M203639200 PMC2930616

[B24] Lozach A. Garrel G. Lerrant Y. Berault A. Counis R. (1998). GnRH-dependent up-regulation of nitric oxide synthase I level in pituitary gonadotrophs mediates cGMP elevation during rat proestrus. *Mol. Cell. Endocrinol.* 143 43–51 9806349 10.1016/s0303-7207(98)00135-x

[B25] McArdle C. A. Franklin J. Green L. Hislop J. N. (2002). Signalling, cycling and desensitisation of gonadotrophin-releasing hormone receptors. *J. Endocrinol.* 173 1–11 11927379 10.1677/joe.0.1730001

[B26] Miyata A. Arimura A. Dahl R. R. Minamino N. Uehara A. Jiang L. Culler M. D. Coy D. H. (1989). Isolation of a novel 38 residue-hypothalamic polypeptide which stimulates adenylate cyclase in pituitary cells. *Biochem. Biophys. Res. Commun.* 164 567–574 2803320 10.1016/0006-291x(89)91757-9

[B27] Mutiara S. Kanasaki H. Oride A. Purwana I. N. Shimasaki S. Yamamoto H. Miyazaki K. (2009). Follistatin gene expression by gonadotropin-releasing hormone: a role for cyclic AMP and mitogen-activated protein kinase signaling pathways in clonal gonadotroph LbetaT2 cells. *Mol. Cell. Endocrinol.* 307 125–132 19533841 10.1016/j.mce.2009.02.030

[B28] Naor Z. (2009). Signaling by G-protein-coupled receptor (GPCR): studies on the GnRH receptor. *Front. Neuroendocrinol.* 30 10–29 18708085 10.1016/j.yfrne.2008.07.001

[B29] Naor Z. Koch Y. Chobsieng P. Zor U. (1975). Pituitary cyclic AMP production and mechanism of luteinizing hormone release. *FEBS Lett.* 58 318–321 178535 10.1016/0014-5793(75)80288-2

[B30] Naor Z. Leifer A. M. Catt K. J. (1980). Calcium-dependent actions of gonadotropin-releasing hormone on pituitary guanosine 3′,5′-monophosphate production and gonadotropin release. *Endocrinology* 107 1438–1445 6253266 10.1210/endo-107-5-1438

[B31] Navratil A. M. Bliss S. P. Berghorn K. A. Haughian J. M. Farmerie T. A. Graham J. K. Clay C. M. Roberson M. S. (2003). Constitutive localization of the gonadotropin-releasing hormone (GnRH) receptor to low density membrane microdomains is necessary for GnRH signaling to ERK. *J. Biol. Chem.* 278 31593–31602 12791688 10.1074/jbc.M304273200

[B32] Oh D. Y. Song J. A. Moon J. S. Moon M. J. Kim J. I. Kim K. Kwon H. B. Seong J. Y. (2005). Membrane-proximal region of the carboxyl terminus of the gonadotropin-releasing hormone receptor (GnRHR) confers differential signal transduction between mammalian and nonmammalian GnRHRs. *Mol. Endocrinol.* 19 722–731 15563546 10.1210/me.2004-0220

[B33] Pawson A. J. Faccenda E. Maudsley S. Lu Z. L. Naor Z. Millar R. P. (2008). Mammalian type I gonadotropin-releasing hormone receptors undergo slow, constitutive, agonist-independent internalization. *Endocrinology* 149 1415–1422 18039780 10.1210/en.2007-1159

[B34] Shacham S. Cheifetz M. N. Fridkin M. Pawson A. J. Millar R. P. Naor Z. (2005). Identification of Ser153 in ICL2 of the gonadotropin-releasing hormone (GnRH) receptor as a phosphorylation-independent site for inhibition of Gq coupling. *J. Biol. Chem.* 280 28981–28988 15964850 10.1074/jbc.M500312200

[B35] Starzec A. Jutisz M. Counis R. (1989). Cyclic adenosine monophosphate and phorbol ester, like gonadotropin-releasing hormone, stimulate the biosynthesis of luteinizing hormone polypeptide chains in a nonadditive manner. *Mol. Endocrinol.* 3 618–624 2542778 10.1210/mend-3-4-618

[B36] Thackray V. G. Mellon P. L. Coss D. (2009). Hormones in synergy: regulation of the pituitary gonadotropin genes. *Mol. Cell. Endocrinol.* 314 192–203 19747958 10.1016/j.mce.2009.09.003 PMC2815122

[B37] Theoleyre M. Berault A. Garnier J. Jutisz M. (1976). Binding of gonadotropin-releasing hormone (LH-RH) to the pituitary plasma membranes and the problem of adenylate cyclase stimulation. *Mol. Cell. Endocrinol.* 5 365–377 824166 10.1016/0303-7207(76)90019-8

[B38] Tsutsumi R. Mistry D. Webster N. J. (2010). Signaling responses to pulsatile gonadotropin-releasing hormone in LbetaT2 gonadotrope cells. *J. Biol. Chem.* 285 20262–20272 20406815 10.1074/jbc.M110.132662 PMC2888439

[B39] Willars G. B. Heding A. Vrecl M. Sellar R. Blomenrohr M. Nahorski S. R. Eidne K. A. (1999). Lack of a C-terminal tail in the mammalian gonadotropin-releasing hormone receptor confers resistance to agonist-dependent phosphorylation and rapid desensitization. *J. Biol. Chem.* 274 30146–30153 10514504 10.1074/jbc.274.42.30146

[B40] Willars G. B. Royall J. E. Nahorski S. R. El-Gehani F. Everest H. McArdle C. A. (2001). Rapid down-regulation of the type I inositol 1,4,5-trisphosphate receptor and desensitization of gonadotropin-releasing hormone-mediated Ca2+ responses in alpha T3-1 gonadotropes. *J. Biol. Chem.* 276 3123–3129 11069921 10.1074/jbc.M008916200

[B41] Winters S. J. Ghooray D. Fujii Y. Moore J. P. Jr. Nevitt J. R. Kakar S. S. (2007). Transcriptional regulation of follistatin expression by GnRH in mouse gonadotroph cell lines: evidence for a role for cAMP signaling. *Mol. Cell. Endocrinol.* 271 45–54 17482756 10.1016/j.mce.2007.03.006

[B42] Zheng L. Stojilkovic S. S. Hunyady L. Krsmanovic L. Z. Catt K. J. (1994). Sequential activation of phospholipase-C and -D in agonist-stimulated gonadotrophs. *Endocrinology* 134 1446–1454 8119185 10.1210/endo.134.3.8119185

